# Analyzing the EU Migration Crisis as Reflected on Twitter

**DOI:** 10.1007/s42489-022-00114-6

**Published:** 2022-07-09

**Authors:** Sagnik Mukherjee, Eva Hauthal, Dirk Burghardt

**Affiliations:** Institute of Cartography, Technische Universität Dresden, 01069 Dresden, Germany

**Keywords:** Twitter, User generated data, EU migration crisis, Geospatial visualization, Social media

## Abstract

The proliferation of social media has resulted in its extensive use as a valuable source of information for researchers. This paper aims to use Twitter data to analyze and visualize tweets about the migration crisis in the European Union from 2016 to 2021. The paper uses a methodology to structure data for better understanding of complex social media data. The methods and metrics include the facet model of location based social media, the HyperLogLog data structure and novel uses of the metric typicality. The authors have also developed a web based interactive application closely following the methodology used to organize the dataset. Additionally the work also includes maps using spatial typicality which could be utilized for studying spatial phenomenon. The case study selected also provides unique insights and sets a template for working with multi-lingual geo-social media data. The authors believe that these methods and metrics could be reproduced for other case studies and aid in understanding and communication geo-social media data.

**Supplementary Information:**

The online version contains supplementary material available at 10.1007/s42489-022-00114-6.

## Abstract (de)

Die Verbreitung sozialer Medien hat dazu geführt, dass sie in großem Umfang als wertvolle Informationsquelle von Forschern genutzt werden. In diesem Beitrag sollen Twitter-Daten von 2016 bis 2021 zur Analyse und Visualisierung von Tweets über die Flüchtlingskrise in der Europäischen Union genutzt werden. Der Beitrag verwendet eine Methodik zur Strukturierung von Daten für ein besseres Verständnis komplexer Social-Media-Daten. Die Methoden und Metriken umfassen das Facettenmodell für ortsbezogene soziale Medien, die HyperLogLog-Datenstruktur und das neuartige Maß der Typikalität. Die Autoren haben zudem eine webbasierte interaktive Anwendung entwickelt, die sich eng an die zur Strukturierung des Datensatzes verwendete Methodik anlehnt. Darüber hinaus enthält der Beitrag Karten, die räumliche Typikalität darstellen und für die Untersuchung raumbezogener Phänomene verwendet werden können. Die ausgewählte Fallstudie bietet außerdem besondere Einblicke in die Thematik und stellt eine Vorlage für die Arbeit mit mehrsprachigen Daten aus geo-sozialen Medien dar. Die Autoren sind der Ansicht, dass diese Methoden und Metriken für andere Fallstudien reproduziert werden könnten und zum Verständnis sowie zur Kommunikation von Daten aus geo-sozialen Medien beitragen.

## Introduction

Social media began as a medium for the global community to connect in an increasingly globalized world. This allowed a unique opportunity for previously local issues to be popularized beyond national and even continental boundaries (Pruthi et al. 2015). One such popular social media network is the microblogging platform, Twitter. Various kinds of socio-political themes have found their way on Twitter, being debated, and retweeted all over the world. Looking back to the last decade, it would be difficult to find a topic which was not discussed on Twitter, not only in English but in many different languages. Twitter has also been closely followed by academics to reap the benefits of this new source of user generated data. But a sizeable proportion of the work has been performed on tweets over a relatively short period of time, mainly as close to real time as possible.

The innovations of this work are, that the authors used tweets over a longer span of time beside the typicality measure, Location Based Social Media (LBSM) structure, the HyperLogLog (HLL) data format and related visualization techniques to make sense of this multifaceted dataset. The case study chosen for this work is the migration crisis in the European Union. The case study offers the complexity of having various languages and is a long-term ongoing crisis. This allows the authors to use the LBSM structure and typicality to set a workflow for making sense of complex datasets. The authors have developed a web-based interactive application in a dashboard format which visualizes the results, using the methods and metrics mentioned above. The authors believe that the LBSM structure, typicality measure and the HyperLogLog format can not only help in understanding social media data but can be further used for structuring visualizations that are effective at scientific communication.

## State of the Art

Hashtags first appeared on Twitter and are coined to be a true child of the internet (Parker 2011). Since then they have spread to many different social media platforms and have a multitude of use cases (Laucuka 2018). Keeping with the spirit various use cases of hashtags, researchers have also studied and opined on a variety of topics related to hashtags, e.g. Hauthal et al. (2019) have used hashtags for mining opinions related to the Brexit referendum. Twitter itself is a platform for political discussions and has been studied widely under Korean, Canadian and other cultural contexts (Choi et al. 2014; Gruzd and Roy 2014; Guerrero-Solé, 2018; Highfield 2013). Twitter hashtags have also been widely researched for the purpose of event detection. Atefeh and Khreich (2015) have reviewed various kinds of event detection techniques on Twitter. Additionally, they have also briefly touched upon the challenges of retrieving retrospective events on Twitter compared to real time events. This paper aims to enrich the research on analysis of retrospective events.

An important part of the analysis is visualization of the twitter data to analyze and detect events. Kumar et al. (2014) have outlined techniques to visualize temporal, geospatial and textual data. Independently, these visualizations will not be able to bring events into focus. Hence they would need to be combined, which made using dashboards an obvious choice. Dashboards are effective visualizations for decision making (Few 2006). But in the case of this work, the dashboard would be a tool for data exploration (Janes A. et al. 2013). Compared to static visualizations, interactive visualizations would allow navigating, selecting and making sense of complex data (Janvrin et al. 2014) and for filtering based on specific conditions (Yi et al. 2007). These features could, potentially be used along with the mentioned LBSM structure introduced in Sect. 3.1 for visualizing the data.

In relation to the migrant crisis on social media, Ferra and Nguyen (2017) analyzed 4277 tweets shared on the 26th of February 2016 reporting that the digital sphere of communication about the crisis is shaped by preexisting hierarchies found in the “offline” sphere. Öztürk and Ayvaz (2018) have worked on English and Turkish tweets related to the crisis. They report more contra sentiments in English tweets as compared to Turkish tweets. Urchs et al. (2019) have used Twitter data from 2015 to create a predicting and extraction system. Inuwa-Dutse et al. (2020) have found greater contra sentiments expressed by unverified Twitter accounts compared to verified accounts on a global dataset of tweets. Hübl et al. (2017) have used geo-tagged tweets to visualize spatial patterns in refugee movement trajectories in Europe. None of these studies have accounted for more than three languages making this work and the methodology of partial opinion analysis in multilingual dataset an interesting opportunity.

## Methods

### LBSM Facet Structure

Reactions to events in LBSM can be described by different facets (Dunkel et al. 2019). The authors define an individual reaction from a single actor to a single event as a reaction that cannot be further differentiated in a meaningful way. They define a reaction as a tuple as (Eq. 1 Definition of a reaction in LBSM)

1 $${\text{r }} = \, \left( {{\text{e}},{\text{ p}}^{{\text{r}}} ,{\text{ t}}^{{\text{r}}} ,{\text{ s}}^{{\text{r}}} ,{\text{ a}}^{{\text{r}}} } \right)$$

where, e stands for the event which is being reacted to.

p^r^ stands for actor who reacted. This is the social facet. The actor here can not only be an individual, but also institutions and organizations. This facet also considers the wider socio-political ecosystem that the actor belongs. LBSM does not directly capture the complex relation of the actor in their ecosystem, but these relations can be inferred from other data like the language, gender, age, etc.

t^r^ stands for the time of the reaction which is called the temporal facet. This facet contains not only the timestamp of the LBSM post, but also when the posts were modified or when the user joined the social media platform.

s^r^ stands for the location of the reaction which is known as the spatial facet. Together with the temporal facet, the spatial facet forms the basis of characterizing a reaction and inferring the event.

a^r^ stands for the for the combination of attributes which define the reaction. This is known as the thematic/topical facet. The topical facet encompasses the situational reaction of the actor. It may include sentiments expressed (Hauthal 2015) or inferring the disposition of the actor from the title, comments or descriptions of the post (Zeng et al. 2016).

The facets not only provide a conceptual model for analyzing LBSM reactions, but also provide abstraction layers for added privacy protection by adjusting the granularity of the data (Löchner et al. 2018).

For the purpose of this work, the tweets obtained are the reactions r. Along with the tweets, Twitter provides the social, temporal, spatial and topical information (p^r^, t^r^, s^r^ and a^r^ respectively). The authors are looking to investigate the events e and to visualize the results with the help of the facets leading to the detected events.

### Typicality

Typicality is derived from the adjective typical which describes the “state of being that is typical”. The formula was developed to combine two relative frequencies: one of a sub-dataset and the other of a total dataset (including the sub-dataset) of emojis or something similar (Hauthal et al. 2021). The typicality *t* is described by (Eq. 2 Definition of typicality)

2 $$t = \frac{{f_{s} - f_{t} }}{{f_{t} }}$$

where, *f*_*s*_ is the relative frequency of the sub-dataset and *f*_*t*_ is the relative frequency of the total dataset. Both frequencies are calculated using (Eq. 3 Definition of relative frequency)

3 $$f = \frac{n}{N}$$

where *n* stands for the absolute number of a specific emoji or hashtag and *N* stands for the absolute number of all emojis or hashtags.

Typicality has either a positive (typical) value or a negative (atypical) value. Compared to other tests for measuring normalization and relative differences, typicality is rather straightforward and easy to comprehend. In addition to this, typicality also delivers more meaningful information because of the sub-dataset in use. The most frequent hashtag or emoji in a dataset, might not be the most typical in the partial dataset which could lead to further interpretations of their use.

### HyperLogLog

The HyperLogLog (HLL) (Flajolet P. et al. 2007) is a solution for the count-distinct problem (Leskovec et al. 2020). The count distinct problem refers to the issue of calculating cardinality in a multiset (Cantor 1895). This multiset could represent anything from unique visitors on a website to motifs in a DNA sequence. HLL is a solution to this problem and is a probabilistic cardinality estimator. The algorithm is able to estimate cardinalities of dataset having more than 10^10^ elements with an error rate of 2%, using only 1.5 kB of memory (Flajolet P. et al. 2007). The functioning of this algorithm can be used for privacy preserving purposes of LBSM data.

The concern amongst users on social media for risks to privacy has increased in recent years. According to a 2014 poll by the Pew Research Center, 91% of the respondents “agree” or “strongly agree” to consumers losing control over how companies collect and use personal information from social media (Madden 2014). In 2020 the European Union Agency for Fundamental Rights published a report in response to governments in the EU discussing the use of technologies to stem the spread of COVID-19. According to this report 41% do not want to share their personal data with companies, which is almost double the number compared to public bodies (European Union Agency for Fundamental Rights 2020).

The benefit of privacy is achieved through multiple steps to prepare the data for analysis. Publicly available Volunteered Geographic Information (VGI) data is converted into the HLL format. This conversion involves using a cryptographic hashing function on the original data to obtain a multiset of binary representations of the original multiset. This binary multiset is divided into numerous multisets to reduce the variance of the final cardinality estimation. From each of these multisets, the maximum leading number of zeros in the binary representation is taken and the harmonic mean of all the leading number of zeros from each of the multisets is calculated to estimate the cardinality of the entire multiset. The privacy aspects are enacted by cryptographically hashing the sensitive information fields found in LBSM data. For Twitter, these fields include user id’s and post id’s. Online Resource 3 provides an example of one such table which contains hashed user id’s and post id’s. The LBSM raw dataset, would have human readable numbers making it quite simple to abuse the data. Additionally, the hashing also uses a secret salt (Kaliski 2000) which substantially improves the security of hashed elements. The detailed software architecture which realizes these steps can be found in Dunkel et al. (2020). Furthermore, increasing spatial granularity of location data by using GeoHash (Dunkel et al. 2020), the format reduces risks by following geoprivacy-by-design recommendations (Kounadi et al. 2018). These techniques also mitigate the risks from intersection attacks by incorporating recommendations (using a secret salt) from Desfontaines et al. (2018).

## Data Processing

### Data Collection and Filtering

There were two main conditions required for the tweets to be relevant for the study; first, the tweets must be geo-tagged, and second, they must be relevant to the migration crisis in EU. The first condition was fulfilled by filtering for tweets with attached longitude and latitude information. For the second condition to be met, hashtags relevant to the migration crisis were used based on a list of relevant hashtags, which the authors requested from the Sächsischer Flüchtlingsrat (SFR), a registered association that campaigns for the interests and rights of refugees and asylum seekers in the Free State of Saxony, Germany. These hashtags were in English and German. Additionally, the authors decided to query hashtags in not only the major European languages (English, German, French, Spanish, Italian, Dutch) but also in the native language of the countries facing the migration crisis. Other than Italy and Spain, a large number of people also come into Europe through Greece and Turkey (Idemudia and Boehnke 2020). Hence the languages chosen for the initial query were: English, German, Italian, Spanish, Dutch, French, Russian, Turkish and Greek.

There are two major reasons for not including languages spoken by refugees into our dataset. Firstly, there is actually a lack of reliable data of what languages the refugees speak. A 2017 report by Translators without Borders^1^ went into details about the assumptions made of languages spoken by refugees. They interviewed 46 humanitarian organizations and found that they did not ask or report on the mother tongue of refugees, thereby contributing to the lack of data. Nigeria, a major country of origin for refugees, is home to 520 first languages. This shows that making assumptions about expected spoken languages on the basis of home countries, is very problematic. Aid workers or government agencies expecting people from Syria or Iraq to speak Arabic, find that the refugees speak Kurdish or other Turkmen languages. The second reason for not keeping languages spoken by refugees in the dataset is that the authors are not familiar in any of the languages stated above. On extracting tweets, the authors could just mistake tweets from non-refugees containing the pre-defined hashtag (which itself will be translated using Google Translate) as hashtags from refugees. Furthermore, Gillespie et al. (2016) have found that Syrian refugees (they could be Kurdish speaking or Arabic speaking) are more tech-savvy than their Iraqi or Afghani counterparts when using social media. This could also paint a picture of all refugees under the same light. Hence the author for the sake of convenience have decided to include only European languages.

Keeping all the above consideration, the data to be collected temporally ranges from 2016 till 2021 and spatially would cover Europe and the northern parts of Africa, filtered using the hashtags relating tweets to the migration crisis. The data was collected using the Twitter API which limits tweets collected to 1% of all tweets being tweeted at the time of the query. Geo-referenced tweets in English only account for 2.17% of all tweets and percentages of all the other queried languages are similarly low (Leetaru et al. 2013). Moreover, (Sloan et al. 2013) opined that the amount of geo-referenced tweets closely follow the geographic population distribution. Therefore, the constraints of the Twitter API were not expected to significantly hinder the work. The results of the query underwent a two-step refinement process. The first was to simply check if the hashtags used for the query returned tweets relevant to the migration crisis. For this step, the co-occurring hashtags were examined. This was necessary due to the query used for retrieving the tweets uses word fragments that can be ambiguous and therefore return tweets that do not relate to the desired topic. An example for that is shown in Code 1.

$${\text{WHERE LOWER}}\left( {{\text{tweet}}\_{\text{text}}} \right){\text{ LIKE }}^{\prime}\% {\text{asyl}}\% ^{\prime}$$

Code 1 Snippet from the initial SQL query for retrieving relevant tweets

The word *asyl* is German for the English word “asylum”. The query in *Code 1* returned tweets containing the word *asyl* in both the tweet body and hashtags. But, since the ‘%’ operator was used, the query additionally returned tweets having conjugations with the root word *asyl.* An example of such a hashtag would be *asylum16*, which does contain the root word *asyl* but the hashtag has no relevance with the migration crisis in EU. Hashtags such as *asylum16* are referred to as the semantically non-relevant hashtags. After this initial query, both semantically relevant and non-relevant hashtags were found in the dataset, leading the authors to a second round of filtering. This was the second step of the aforementioned two-step refinement process.

After the removal of the semantically irrelevant hashtags, the new relevant co-occurring hashtags were included in a second query. These co-occurring hashtags were selected based on the number of times they appeared in the first query and were absent in the initially decided upon list of hashtags. For example, the hashtag *asylrecht* appeared as a co-occuring hashtag in the initial query but was not included in the list of queried hashtags. Therefore this hashtag was included for the second round of querying, after removing the semantically non-relevant hashtags. At this point, the hashtags in Turkish, Greek and Russian were also excluded as the low number of tweets returned in the initial query did not justify their inclusion in the dataset. Furthermore, the non-Latin script of these languages posed unique challenges while performing the semantic filtering described above. After these refinement steps, a second query was conducted which returned approximately 170,000 tweets from February 2016 until January 2021, posted within the bounding box of Europe. This was used as the final dataset for the study.

### Data Storage and Preparation

The entire dataset was stored on two separate PostgreSQL servers, one server for the raw tweets and the other for the tweets in HLL format. Here the term “raw tweets” refers to tweets that have been converted into LBSM raw format by using the program lbsntransform.^2^ The methodology of exploring both formats of tweets followed the LBSM structure (Dunkel et al. 2019). There are, however, particularities involved in exploring the raw tweets and HLL format tweets which are explained below.

The tweets in the privacy aware data format are divided along the four facets. Each of the four facets have their own schema in the HLL server besides tables with various facets of data. The analysis was performed mostly on separate Jupyter Notebooks by downloading CSV’s from the tables and then working with the data.

The primary methodology of exploring HLL data is based on union and intersection functions which combine to form the inclusion–exclusion principle. Equation 4 shows the inclusion–exclusion principle for two sets and Eq. 5 for three sets respectively (Eq. 4 Inclusion–exclusion principle involving two sets, Eq. 5 Inclusion–exclusion principle involving three sets).

4 $$|{\text{A}} \cup B\left| = \right|{\text{A}}\left| + \right|{\text{B}}\left| - \right|{\text{A}} \cap {\text{B}}|$$

5 $$|{\text{A}} \cup {\text{B}} \cup {\text{C}}\left| = \right|{\text{A}}\left| + \right|{\text{B}}\left| + \right|{\text{C}}\left| - \right|{\text{A}} \cap {\text{B}}\left| - \right|{\text{A}} \cap {\text{C}}\left| - \right|{\text{B}} \cap {\text{C}}\left| + \right|{\text{A}} \cap {\text{B}} \cap {\text{C}}|$$

Using the HLL union and intersection function and the inclusion–exclusion principle, it is possible to explore the HLL sets. For example, considering tweets associated with the hashtag *refugeeswelcome* as set A and tweets associated with another hashtag like *migrants* as set B, Eq. 4 could be used to find total tweets containing both hashtags. In the case of three hashtags, Eq. 5 would be suitable. A complete example, using both the aforementioned equations, is depicted in the supplementary materials submitted along with this work (Online Resource 2). This process can also be recursively iterated in case if associations with more than three hashtags are to be investigated. The result of an initial intersection between A and B could be also intersected with the results of an intersection between set C and set D. Therefore, the HLL functions allow for flexibility in qualitative analyses.

**Fig. 1:**
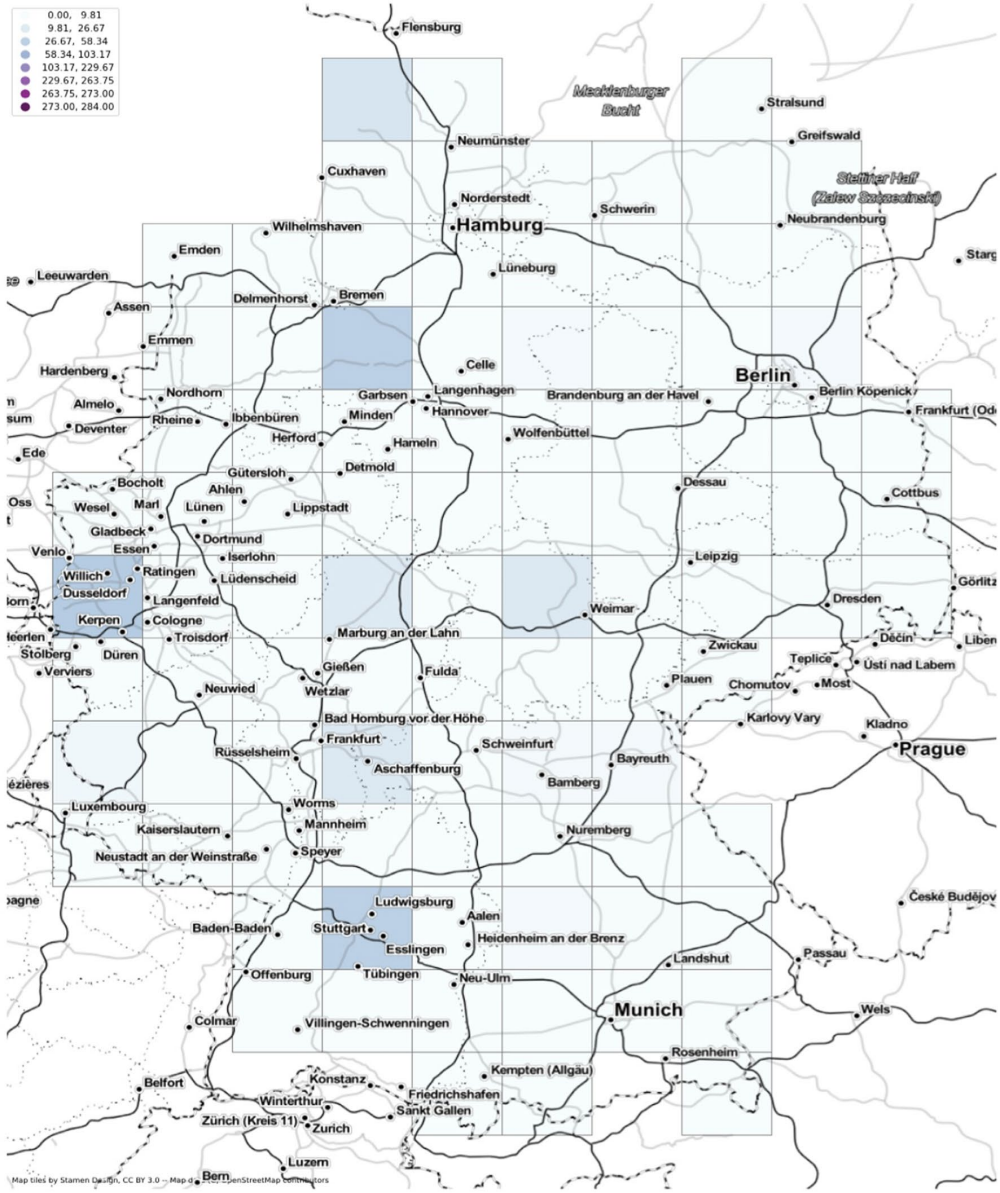
Illustration of visualizing HLL data (user_count) with geohashed latitude and longitude

Visualizing the HLL data involves the use of geohashing (Niemeyer 2008) which is a kind of Z order curve (Morton 1966). Geohashing was developed for efficient storage of large amounts of geographic coordinates by sacrificing the precision of the co-ordinates. In the case of HLL data format, visualizing geohashed coordinates adds a further level of security. The individual latitude and longitude data obtained after geohashing are further aggregated into grid cells. Depending on the values within each individual grid cell, the cells are colored to make a choropleth map. Having a basemap along with the grids adds a spatial context and improves data readability. Figure 1 depicts an example illustration of the final map after performing the aforementioned processing.

The raw data, stored in a different PostgreSQL server came with several different columns. Not all the columns were used for this work as they were not related to the facets of LBSM.

The spatial facet, consisting of the latitude and longitude of the tweets, were stored in the server in the Well Know Text (WKT) format (Herring 2011). Their re-projection and extraction into latitude and longitude points were made through the ST_TRANSFORM, ST_X and ST_Y functions in POSTGIS. During this time, it was also found that the second step of semantic filtering described in Sect. 4.1 has not been sufficient. Due to the presence of the hashtags *deport* and *moria*, various conjugations of the Spanish word *deportistas* like *deportivo* were found in the database. Occurrence of the English hashtag *moriarty* was also found. These hashtags are also semantically non-relevant hashtags but were not filtered during the two-step filtering previously described. They were consequently excluded from the third query result (made in the PostgreSQL server) to retrieve all tweets into a comma separated value (csv) file for further analysis on Jupyter Notebooks. The percentage of erroneous hashtags was not more than 2% in this case.

The raw tweets in the csv needed some formatting to be effectively visualized and interpreted. This formatting was dependent on the facet of the data. A short summary of the formatting of the data according to the facets is shown in Table 1.

**Table 1:** Summary of data formatting according to the facets

Facet	Formatting
Spatial	No formatting required
Temporal	Aggregation of timestamps into months/years
Topical	Removing braces, quotation marks, turning all letters into lower case
Social	Not used for the raw tweets

For the temporal facet, the raw tweets were provided with timestamps in the format of YYYY:MM:DD HH:MM:SS. As previously mentioned, the dataset for this work spanned for five years from 2016 to 2021. It was then decided to focus on aggregating monthly or yearly timestamps instead of daily cycles. Furthermore, considering the migration crisis, better results would be expected from monthly aggregation as compared to daily or hourly aggregations. This was reasoned based on the nature of the crisis which is long term as compared to a flooding event which has a shorter temporal duration and therefore requires more fine-granular data.

For the topical facet, there were two major columns in the raw dataset to be considered for the analysis: hashtags and the tweets which echoes the main thrust of this work.

### Facet Exploration Tool

With the individual facets or even with a combination of multiple facets, it was required to have a visualization where users could be able to inspect the visualizations simultaneously. Furthermore, while exploring such a diverse dataset, it would be necessary to implement interactivity. Interactivity would allow the users to choose options that they wish to see and would help in presenting the data. Some information about the facets which could not be directly integrated in the static visualizations, could be shown through tooltips. Based on these reasons, the authors decided to implement a facet exploration tool which is close to dashboard to accommodate all the needs.

**Fig. 2:**
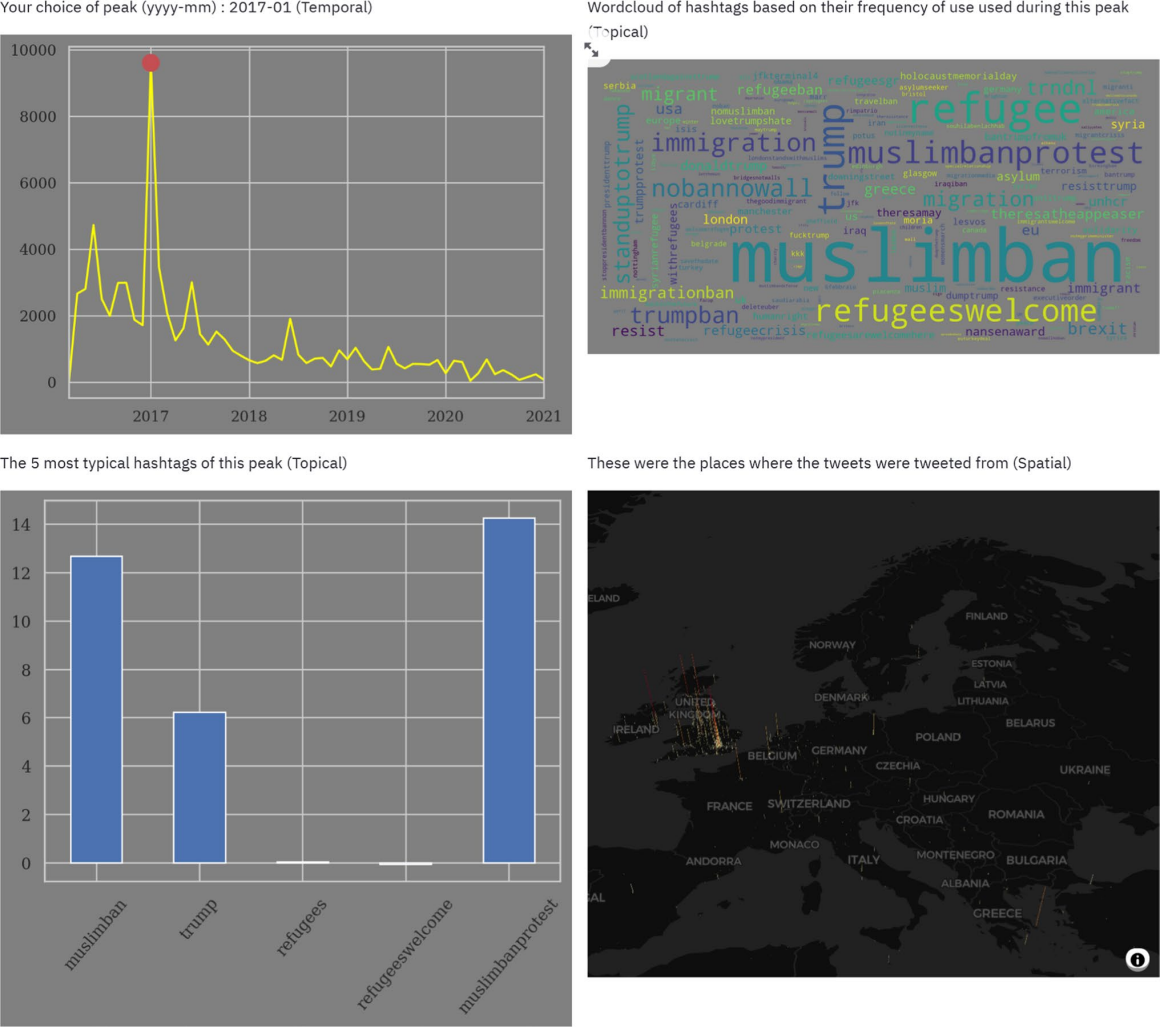
Screenshot of the contents of the events expander taken from the preliminary facet visualization tool

The final tool functions not only as a tool for visualizing the dataset but also as a tool to help in the analysis. An introduction to the facets using the elements of narrative visualization was intended to help non-experts ease themselves into the data by defining the context of the data. This was primarily introduced in the section “Explore the Facets”. In the section “Explore Events with the Facets” (shown in Fig. 2), users both experts and non-experts can use the facet exploration tool to explore the dataset through the events. However, the authors are yet to undertake usability tests to ascertain the effectiveness of the implemented methods. Hence, a detailed discussion of this tool, and the suitability of such a dashboard will be a matter for future work.

## Data Analysis and Interpretation

The visualizations of the individual facets were instrumental in the analysis of the raw dataset in search of opinions and events. The facet exploration tool made was also based on these graphs. But using the visualizations to infer events and to acquire useful knowledge requires multiple graphs of the facets to be examined simultaneously. Additionally, the hashtags themselves must also be investigated along with the multiple visualizations.

According to the dataset, most hashtags from this dataset have only been found in one tweet. Out of the total number of non-unique 68,216 hashtags, 23,632 hashtags have been used more than once in tweets and 14,481 hashtags have been used more than twice in the five years span of the dataset.

Table 2 shows the most popular hashtags across the five years span of the dataset.

**Table 2:** Most popular hashtags

Hashtags	Counts
*Refugees*	16,456
*Migranti*	14,233
*Migrants*	12,282
*Refugeeswelcome*	11,789
*Muslimban*	10,789
*Migration*	8852
*Immigration*	7454
*Refugee*	4562
*Refugiados*	3756
*Immigrazione*	3082

One reason for such a distribution of the hashtags could be the use of hashtags during specific events. Some hashtags like *refugees* or *refugeeswelcome* are primarily used to mark the tweet as something relevant to the topic of the migration crisis. The other hashtags become more context specific, for example *muslimban* which is used specifically to denote one particular event. In addition to hashtags like *refugees,* other hashtags can also be used to denote a specific sub-event like the World Refugee Day.

**Fig. 3:**
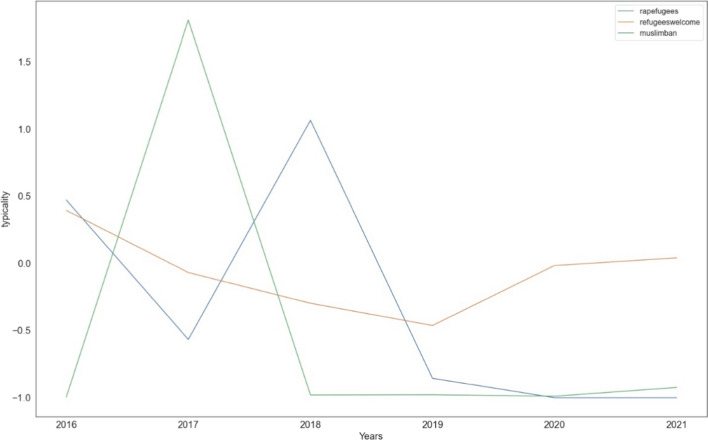
Temporal typicalities of *muslimban*, *refugeeswelcome* and *rapefugees* for every year in the studied dataset

On further exploring these hashtags along the temporal facet, it is possible to detect events. For this purpose typicality was used, by taking a temporal sub-dataset of one year to find hashtags that were typical throughout the years. The temporal typicalities of the hashtags relevant to the forthcoming examples are shown in Fig. 3. Interestingly, the most widely used hashtags for particular languages, like *migranti* in Italian or *refugees* in English can be used to infer temporal windows of heightened tweets. In other words, ff the hashtag is used constantly over a long period with high frequency, the temporal typicality of the hashtag can be used for event detection, as it sharpens periods of significance.

The hashtags have also been explored spatially using typicality. The approach is to divide the custom made Europe shapefile (Online Resource 4) into 100 km × 100 km cells. Each of these cells serve as the sub-dataset while the total number of hashtags serve as the total dataset. When a particular hashtag is not found within a grid (a value of -1.0), it is marked in bright orange. For that grid, the specific hashtag would be atypical. On the other hand, values above 0.50 are marked in green, and they are typical. The details of these calculations can be found in the supplementary materials (Online Resource 1).

### Hashtag *Muslimban*

**Fig. 4:**
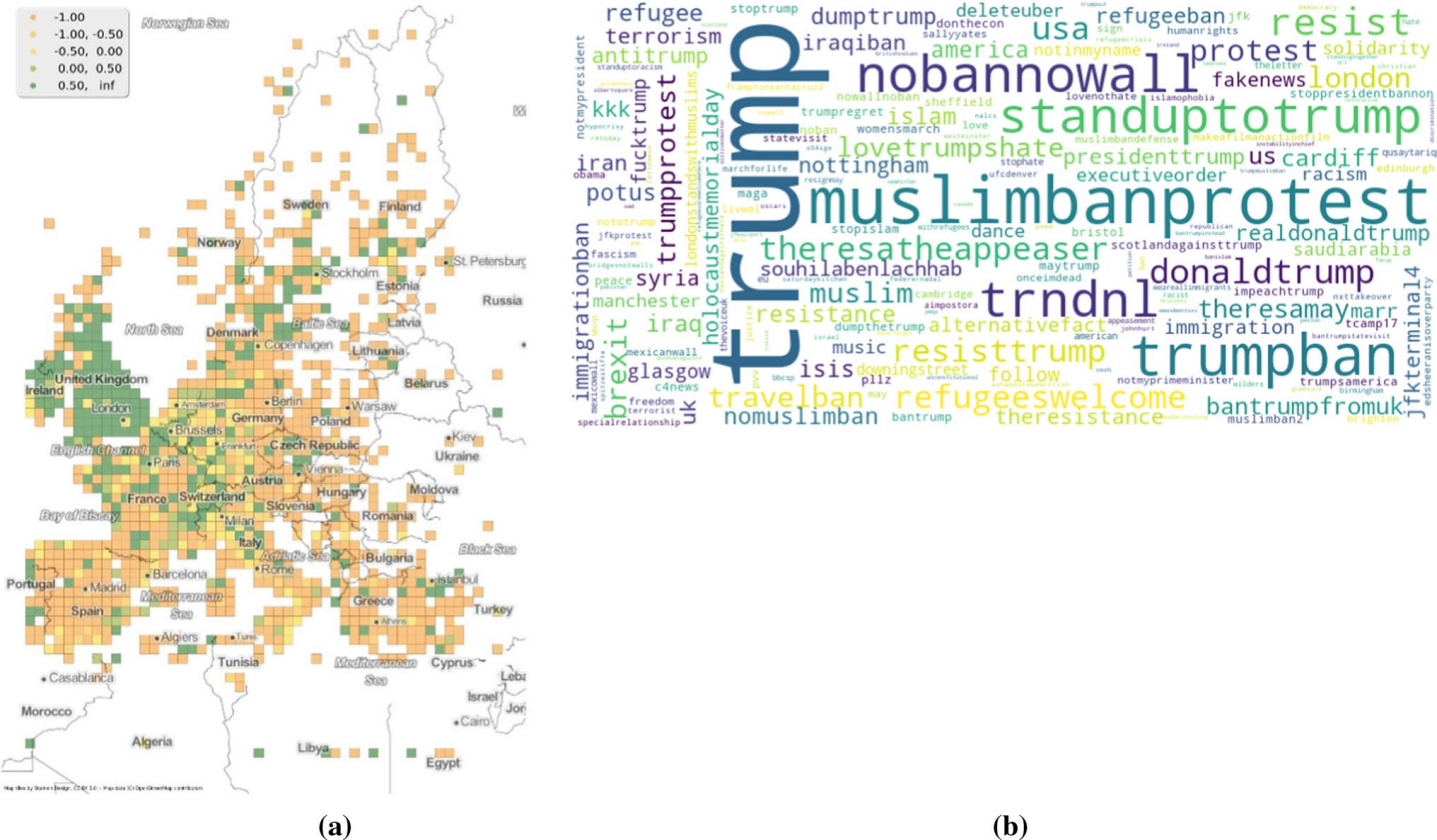
Figure *muslimban* **a** spatial typicality **b** co-occuring hashtags

To demonstrate the use of co-occurring hashtags to pin point specific events and further be useful for opinion analysis, the hashtag *muslimban* is examined in the following section*.* Using typicalities, the temporal window when the hashtag was important can be identified, which is the year 2017. This is shown in Fig. 3. Figure 4b shows the co-occurring hashtags of *muslimban.* Using these two charts, it is possible to connect the tweets shared to President Donald Trump’s signing of the executive order, preventing people of Muslim faith from entering the United States of America (Protecting the Nation from Foreign Terrorist Entry into the United States 2017). If needed, it would have also been possible to look at the monthly typicalities of *muslimban* in 2017 to connect the date of this announcement with the dates of tweets posted. But for this particular case the co-occurring hashtags overwhelmingly point to the aforementioned event.

Interestingly, the event mentioned has nothing of consequence in Europe. It is an event connected to the policy of the United States. From Fig. 4a, the spatial typicality of the hashtag points towards its use within English speaking populations. The dataset used for this work has 41% percent of tweets in English, which explains the prevalence of the hashtag in the United Kingdom. Furthermore, looking at continental Europe, the hashtag is largely used close to urbanized areas. Again, it is well established how Twitter is more prevalent among urban population rather than rural population (Brenner and Duggan 2013). However, this spatial distribution might also indicate the prevalence of Twitter users in Europe and their interests in politics in the United States.

The co-occurring hashtags of *muslimban* additionally might indicate the opinions of users of the hashtags along with the event. Some of the most frequent hashtags used as seen in Fig. 4b are *nobannnowall, muslimbanprotest, trumpban and standuptotrump.* All of these convey a rejection of the proposed measure signed by Mr. Trump and a solidarity for the plight of refugees.

### Hashtag *Refugeeswelcome*

**Fig. 5:**
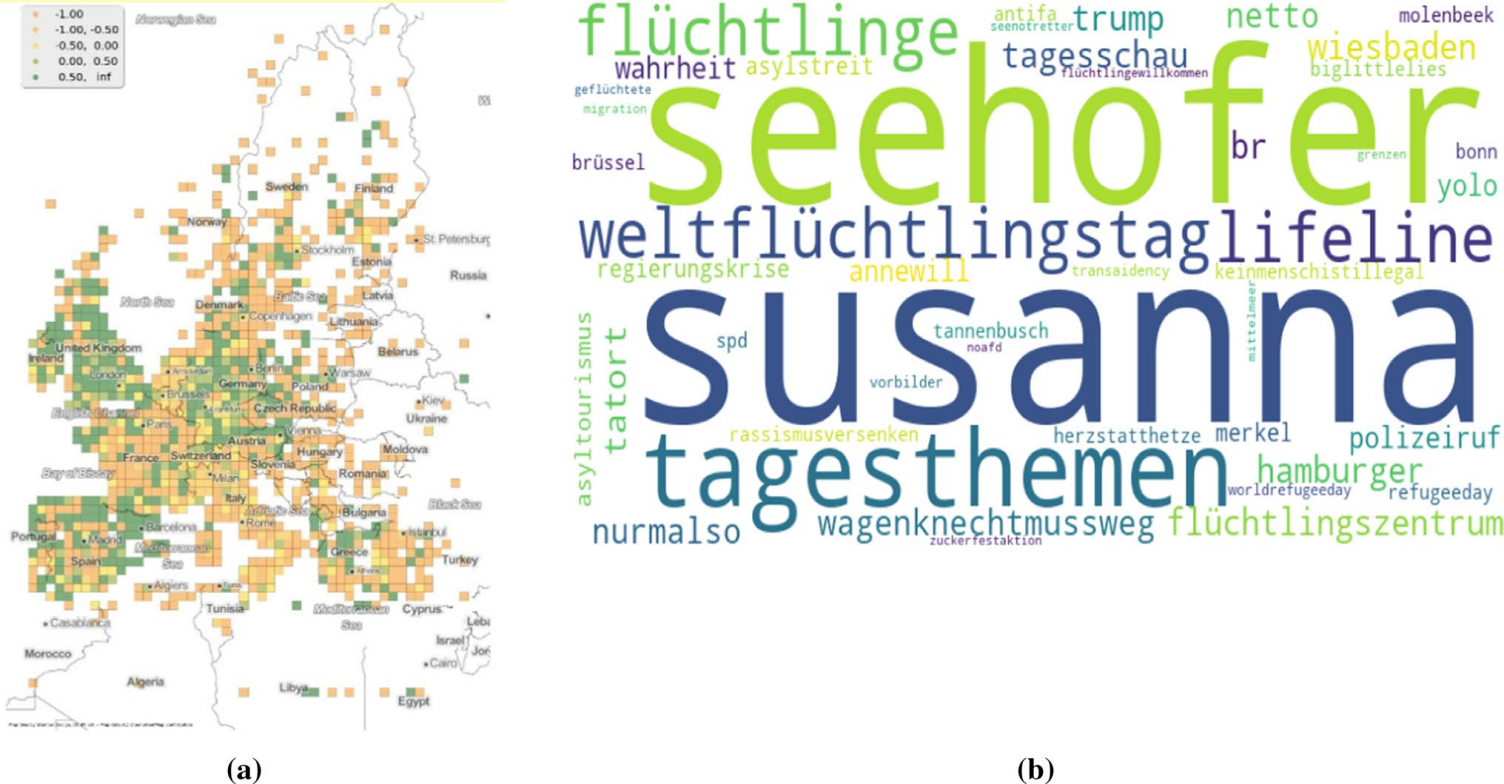
Figure *refugeeswelcome* **a** spatial typicality **b** co-occuring hashtags in July, 2018, Germany

To continue with the approach presented with *muslimban,* the co-occurring hashtags are referred to for an indication of the opinions expressed by the users. However, this time to reduce the effect of the overwhelming prevalence of English, Fig. 5b shows co-occurring hashtags of *refugeeswelcome* used in Germany during the month of July, 2018. The reason for selecting this particular date is due to it being a peak on the German tweets trendline. It was during this time that the Angela Merkel government in Germany was going through a political crisis (Nielsen 2018). The hashtag *refugeeswelcome* might indicate a support towards the cause of the refugees. This conclusion does require some careful consideration specially because *refugeeswelcome* could refer to the movement in Europe to allow refugees to live with European citizens rather than in camps (Refugees Welcome International 2021). The typicality of *refugeeswelcome* also coincides with the years when the movement was popular (Fig. 3). Both hashtags *weltflüchtlingstag* and *flüchtlinge* does lend support to a pro, i.e. supportive interpretation of the hashtag. On the other hand the most frequently used hashtag was *susanna*. This would be a reference to the rape and murder of a 14 year old girl by a 21 year old asylum seeker (VRM GmbH & KG, Co. 2021). Such a situation leads to an ambiguity in the interpretation of the primary hashtag being investigated. This point will be addressed in more details in Chapter 6.

Figure 5a shows the spatial typicality of the hashtag. Its distribution all across Europe comes as no surprise because of it being in English and also because the movement itself was very popular in many European countries. The point to note here is its presence in southern Spain and in Greece. The reasons for these will be discussed in Sect. 5.4.

### Hashtag *Rapefugees*

**Fig. 6:**
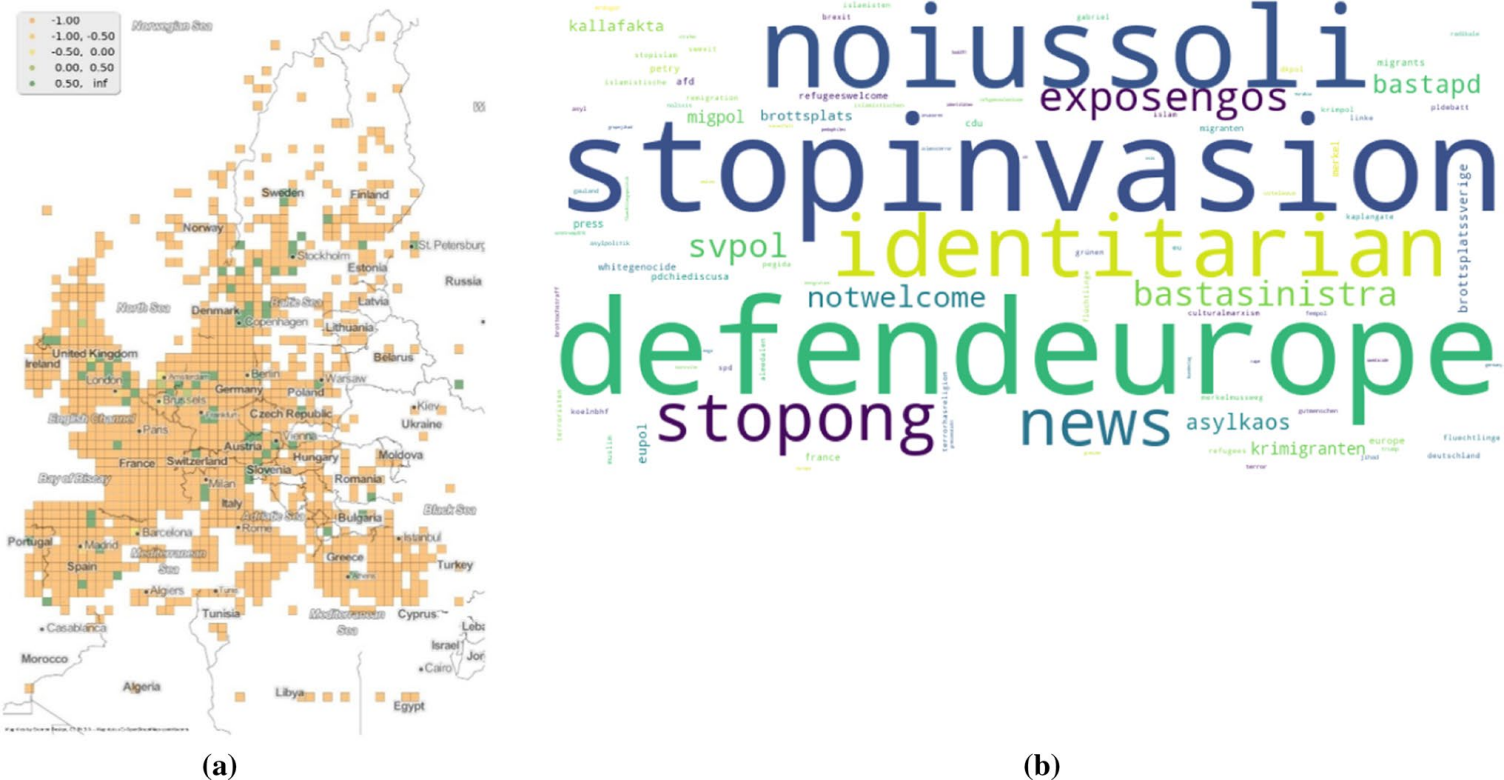
Figure *rapefugees* **a** spatial typicality **b** co-occuring hashtags in German tweets

In the previous two cases, co-occurring hashtags have been used to interpret solidarity (*muslimban*) and ambiguity (*refugeeswelcome*). This clustering of hashtags of solidarity is also found on the contra spectrum of opinions as can be demonstrated with the hashtag *rapefugees* (Fig. 6b). From the co-occurring hashtags of *rapefugees* there is a clear relation to opinions expressed against refugees and immigration in general. The hashtag *identitarian* refers to a pan-European far right group (Mudde 2019). The hashtag *defendeurope* refers to the ship that the Identitarians had chartered to stop migrants from coming to Europe (Walsh 2017).

The temporal typicality of *rapefugees* (Fig. 3) show us that the hashtag was widely used in 2016 and 2018. In the year 2018, one in four Europeans were voting for a populist government (Henley 2018). Furthermore, the spatial typicality of the hashtag (Fig. 6a) does also show their use spatially. Of course the number of hashtags shared is significantly lower in comparison to both *refugeeswelcome* and *muslimban*. The potential reasons for the low number are discussed in Chapter 6.

### Interpretation

There are two distinct observations that are noticeable in the entire dataset. The first one being the peaks having correlations with political events in their respective countries. What was especially interesting was the wide range of popularity of the hashtag *muslimban* in Europe. However the popularity of the hashtag amongst so many languages, does not quite reflect its relationship to the European migration crisis. An explanation of this point is better served by first clarifying the second observation.

**Fig. 7:**
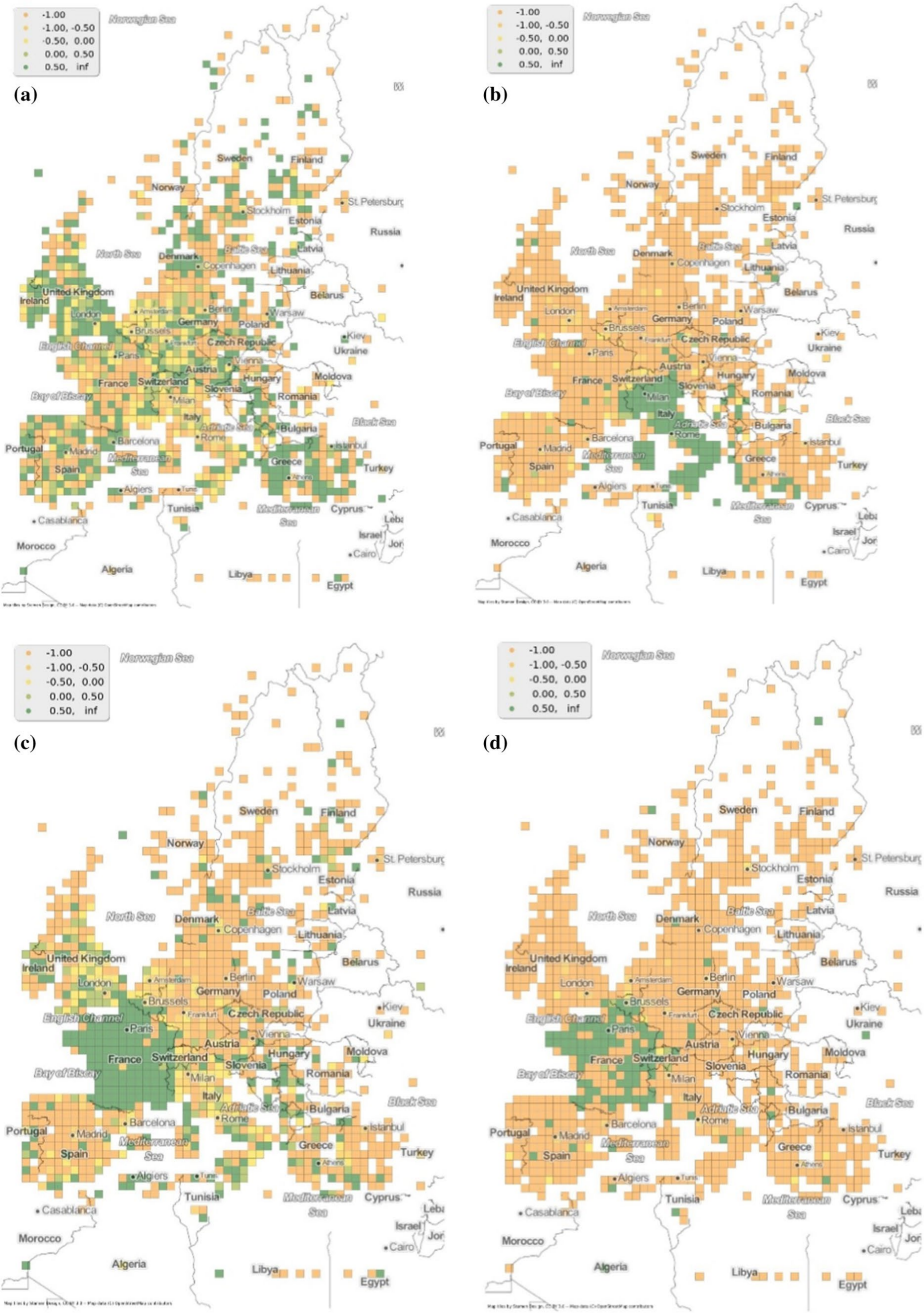
Spatial typicality **a*** refugees ***b** *migranti* **c** *migrants* **d*** réfugiés*

The second observation is made possible due to the spatial typicality of the hashtags. In addition to *refugeeswelcome* and *muslimban,* the hashtags *refugees, migranti* and *migrants* are the five most shared hashtags in the dataset (Table 2). Barring *muslimban,* all the other hashtags are quite typical in the three migration routes to Europe (Idemudia & Boehnke 2020). In fact the spatial typicality of the hashtag *réfugiés*, shows a higher typicality in the north, where Calais is located (Taylor 2021). These maps can be seen in Fig. 7.

The presence of higher twitter activity with regards to the location of major crossing points along the route to destinations of the migrants has also been shown by Hübl et al. (2017) using hashtag clustering. This work uses hashtag typicality and also has more tweets and languages compared to the work previously mentioned. *Muslimban*, in this regard stands out from the other hashtags. Temporally, *muslimban* is typical only in 2017 and spatially is not as widely used in the places under discussion. Therefore, not only is the hashtag *muslimban* topically different from the other hashtags (in not being directly related to the migration crisis) but also spatially and temporally different.

## Discussion and Conclusion

The contribution of this work is broadly in two areas. Firstly, using the facet structure, typicality measure and hashtags for event detection, partial opinion analysis and exhibiting the potential of using them to structure and understand social media data. Secondly using the methods to make visualizations, both independently and in combination (the facet exploration tool) to make interpretations for knowledge production. In short, the combination of these techniques could be used in other case studies for structured data exploration. Additionally using the privacy aware format, which also is based on the facets of LBSM, for exploring and visualizing the data. However, all these methods of exploration do have their inherent advantages and disadvantages.

For the visualization of the HLL data format, the spatial typicality maps in Sect. 5 show the potential of using grids and an underlying base map to add further context to the data while also protecting the privacy of the users. At this point there should be a brief discussion of the modifiable area unit problem (MAUP) (OPENSHAW 1981) with respect to the spatial typicality maps. For the maps presented above, 100 km × 100 km grid cells were used and were considered to the sub-dataset for the calculations. Of course, a larger grid would directly influence the typicality values and for the time being the authors have taken these grid sizes arbitrarily. The authors intend to use the spatial typicality algorithm on further datasets and experiment with grid sizes with respect to the volume of posts for future works.

Despite the advantages outlined above, the authors must also concede to the challenges of using the HLL format for an extensive data exploration. The structure of the dataset after being transformed from the raw tweets to the HLL format is significantly altered. In the raw dataset it is possible to seamlessly switch between the facets as the dataset contains the location, timestamps, and the hashtags together. In the HLL dataset, there are custom made tables containing either a combination of one or two facets. For example, the dataset used for the spatial typicality maps is such an HLL table (Online Resource 3). The columns of the table are aggregated from various other tables in the HLL database of the Postgres server. Calculations for spatial typicality did not include the inclusion–exclusion principles but their use is shown in Online Resource 2. The cumbersome nature of the HLL data does not outweigh the benefits of fast calculations and privacy-aware nature that the data structure inherently offers. The authors would themselves indulge and additionally would like to encourage further research using this data format to improve upon the existing tools for commonplace use of the HLL data structure.

Moving on to the raw dataset, the number of tweets collected by the authors spanning five years and six major European languages was small. This made making definitive conclusions about the opinions of the users quite challenging. The authors wish to explicitly state that the insights and analysis of this work cannot be considered as definitive reactions to the migration crisis. The number of tweets analyzed, the methodologies adopted (like selecting the most frequently used hashtags for typicality analysis) and the lack of reliable comparisons of results severely hinder attempts to make conclusions about reactions to a dynamic and complicated phenomenon. Along with this Twitter only represents a small urban, educated and relatively young part of the demographic. This also means that the reactions on Twitter are only the opinions of certain sections of society and should not be interpreted otherwise. What the authors wish to communicate through this work are the merits and shortcomings of the employed techniques on a linguistically challenging, spatially and temporally broad dataset. Furthermore, using these techniques could be a potential workaround, or at least fill the gaps that current sentiment analysis techniques are unable to fill.

Sentiment analysis was initially attempted by the authors in the hope of broadening of the results to have a more holistic understanding of the dataset. Of the two broad approaches to sentiment analysis, lexicon based and machine learning based (Bhuta et al. 2014), the authors used a lexicon based python library called VADER (Hutto and Gilbert 2014) on the English tweets only. The results of the analysis were not included in this work and the authors decided not to pursue sentiment analysis further based on a few reasons. Primarily cross-language lexicon based sentiment analysis suffers from numerous issues. The authors of GerVADER (Tymann et al. 2019) go into details for the underwhelming performance of their library when trying to adapt VADER for German tweets. Machine learning based approaches could also face problems because of the small size of datasets. (Kruspe et al. 2020) have discussed that their dataset size of 4.6 million tweets helps avoiding random fluctuations of sentiments. These random fluctuations were seen in the results obtained from English tweets of this work, which is why the authors chose not to include them in the paper. Besides these primary reasons, there remains numerous issues (like selecting corpuses, selecting a proper labelling model and comparing results of multi-lingual analyses) when trying to apply sentiment analysis. Hence, it is being attempted by the authors to use the techniques described to be a potential workaround.

The workaround however, inherently has its own disadvantages. These disadvantages can be better explained by considering the co-occurring hashtags of *refugeeswelcome* in German. The presence of the hashtag *susanna* could undermine the interpretation of the pro use of *refugeeswelcome*. Furthermore, the co-occurring hashtags with *susanna* do not clearly give us an answer about the nature of the usage of this hashtag. On the other hand, without the spatial facet filtering for *refugeeswelcome* in Germany, the co-occurring hashtag *susanna* would not been detected. This demonstrates the benefits of using the LBSM structure and the need for contextual information in interpreting the hashtags.

Further plausible questions with regards to ambiguity could also be asked. Again, in the case of *refugeeswelcome* in German, how frequent should the co-occurring hashtags be to denote the primary hashtag to be a pro (or contra depending on the case) opinionated hashtag. For this paper, the authors depended on the presence of the co-occurring hashtags in the word cloud of the primary hashtag to make the aforementioned conclusions. Due to the multilingual nature of the dataset, a sentiment analysis becomes a challenge. In future, the authors hope to improve upon the presented methodology by applying them to multiple case studies. This dataset had more pro hashtags compared to contra hashtags. Adding to that, is the overall small number of 170,000 tweets. Both these factors could be contributing to the ambiguity of the interpretation.

The small number of contra opinionated hashtags could be explained by the use of hashtags from organizations like SFR or United Nations High Commissioner for Refugees (UNHCR) which are pro-refugee institutions. The other reason could be Twitter’s policy against hate speech which automatically removes hostile tweets or hashtags, forcing people with such views to other social media platforms. In this regard topic modelling could be used to improve the accuracy and number of tweets. Latent Dirichlet Allocation (Blei et al. 2003) is a fairly popular algorithm for extracting topics from documents and has been used in the context of social media for extracting posts. But Biterm Topic Model might be better suited for tweets (Jónsson and Stolee 2015). An ideal workflow should probably involve the use of both topic modelling and hand selection used for this work. This should improve the accuracy of the tweets removing semantically non-relevant tweets and improving the total amount of tweets at the same time.

Notwithstanding the drawbacks, the method of analysis employed in this work has provided useful information about the dataset. One of the surprising ones was the popularity of the hashtag *muslimban* across all the languages. Table 2 clearly indicates the popularity of the hashtag and this popularity is detectable across all the major countries of the dataset. This is noteworthy as the event being referred to, is not related to Europe but to the United States. Also, other than the use of *muslimban*, there were few other hashtags which were widely used in all the languages of the dataset. One example would be *withrefugees* which is used by the UNHCR. This naturally gives the hashtag a wider linguistic spread as the United Nations is an international organization. The popularity of *muslimban* could be explained in part by the sheer number of Twitter users in the United States. This already skews the tweets towards having Anglophone content being widely shared compared to other languages. This could also explain a much higher number of English tweets as compared to other languages.

The example of both the hashtag *susanna* and *muslimban* highlight the need for contextual information. This information can be visualized using the facet exploration tool as it allows for looking at every facet of the data simultaneously. The LBSM facet model could also be used to structure the data and make analyses based on this structure. However, a shortcoming of this work is the absence of usability tests to determine the effectiveness of the visualizations and if this visually structuring the data based on facets used, had an overall impact on the presentation and cognition of the data. This is a future work that the authors wish to undertake.

Additionally, the authors would like to focus on refining the methodology applied for this work. By generalizing rules to determining pro and contra hashtags, integrating topic modelling for querying and filtering the dataset, improving the usability of the HLL format and testing the usability of the facet exploration tool, the authors believe that LBSM structure, typicality and the visualizations used for this case study could be invaluable on multiple other cases for knowledge production.

## Supplementary Information

Below is the link to the electronic supplementary material.

Supplementary file1 (IPYNB 683 KB)

Supplementary file2 (IPYNB 534 KB)

Supplementary file3 (CSV 42007 KB)

Supplementary file4 (CPG 0 KB)

Supplementary file5 (DBF 14 KB)

Supplementary file6 (PRJ 0 KB)

Supplementary file7 (SHP 29 KB)

Supplementary file8 (SHX 0 KB)

Supplementary file9 (HTML 969 KB)

Supplementary file10 (HTML 820 KB)

## Footnotes

1 https://translatorswithoutborders.org/wp-content/uploads/2017/04/Putting-language-on-the-map.pdf.

2 https://lbsn.vgiscience.org/lbsntransform/docs/.
