# Supplementary material for: Analyzing the EU Migration Crisis as Reflected on Twitter
Source: KN J Cartogr Geogr Inf. 2022 Jul 9;72(3):213–28. doi: 10.1007/s42489-022-00114-6 (PMC9469823; doi:10.1007/s42489-022-00114-6)
Supplement: Supplementary file 9 — Supplementary file9 (HTML 969 KB) [file 42489_2022_114_MOESM9_ESM.html]

ESM\_1


# Analyzing the EU Migration Crisis as reflected on Twitter¶

### Jounral of Cartography and Geographic Information, Kartographische Nachrichten¶

Author Information

- **Sagnik Mukherjee** : sagnik.mukherjee1@tu-dresden.de ; https://orcid.org/0000-0001-8938-6154
- **Eva Hauthal** : eva.hauthal@tu-dresden.de ; https://orcid.org/0000-0001-8917-600X
- **Dirk Burghardt** : dirk.burghardt@tu-dresden.de ; https://orcid.org/0000-0003-2949-4887

Institute of Cartography, Technische Universität Dresden, 01069 Dresden, Germany

In [1]:

```
#imports

import warnings
warnings.filterwarnings("ignore")
from pathlib import Path
import geopandas as gp
import pandas as pd
from pyproj import Transformer, CRS, Proj
from shapely.geometry import shape, Point, Polygon
from matplotlib.colors import LinearSegmentedColormap
import numpy as np
import shapely.speedups as speedups
import contextily as ctx
from collections import Counter
import matplotlib.pyplot as plt
import mapclassify as mc
speedups.enable()
```

In [22]:

```
"""
Defining constants to be used throughout the program

"""

#create grids based on the custom made eu shapefile

GRID_SIZE_METERS = 100000 
                        
# target projection: Web Mercator (epsg code)
EPSG_CODE = 3857
CRS_PROJ = f"epsg:{EPSG_CODE}"

# Input projection WGS 84
CRS_WGS = "epsg:4326"

# define Transformer ahead of time
# with xy-order of coordinates
PROJ_TRANSFORMER = Transformer.from_crs(
    CRS_WGS, CRS_PROJ, always_xy=True)

# also define reverse projection
PROJ_TRANSFORMER_BACK = Transformer.from_crs(
    CRS_PROJ, CRS_WGS, always_xy=True)

#projecting the bounds of the eu-shapefile to web-mercator

XMIN = PROJ_TRANSFORMER.transform(-9.4203375, 22.33177875)[0]
XMAX = PROJ_TRANSFORMER.transform(35.24322, 22.33177875)[0]
YMAX = PROJ_TRANSFORMER.transform(48.760877, 69.504585)[1]
YMIN = PROJ_TRANSFORMER.transform(48.760877, 28.017169)[1]


#hashtag to be used for typicality calculations
HASHTAG = 'lesbos'

#creating a custom colour map to be used for plotting
colors = ['darkorange', 'gold', 'darkgreen']
CMAP = LinearSegmentedColormap.from_list('mycmap', colors)
```

In [3]:

```
"""
Reading the input data : the dataset of hashtags and the custom shapefile of EU
"""

# using the columns latitude, longitude and hashtags from the entire table

df = pd.read_csv(Path.cwd().parents[0]/ "Data"/ "Spatial_Typicality" / "ESM_3.csv", usecols = [3,4,5]) 
gdf = gp.GeoDataFrame(df,geometry =gp.points_from_xy(df.longitude,df.latitude),crs =CRS_WGS)
eu = gp.read_file(Path.cwd().parents[0] / "Data" / "Spatial_Typicality"/ "ESM_4.shp")

# converting to web-mercator for plotting with contextily
eu.to_crs(CRS_PROJ,inplace =True)
gdf.to_crs(CRS_PROJ,inplace =True)

# take a look at the geodataframe
gdf
```

Out[3]:

|  | latitude | longitude | hashtag | geometry |
| --- | --- | --- | --- | --- |
| 0 | 28.017169 | 1.664273 | afrique | POINT (185266.079 3251138.580) |
| 1 | 28.017169 | 1.664273 | alger | POINT (185266.079 3251138.580) |
| 2 | 28.017169 | 1.664273 | algeria | POINT (185266.079 3251138.580) |
| 3 | 28.017169 | 1.664273 | algerian | POINT (185266.079 3251138.580) |
| 4 | 28.017169 | 1.664273 | algerie | POINT (185266.079 3251138.580) |
| ... | ... | ... | ... | ... |
| 211547 | 70.071230 | 19.437217 | refugeeswelcome | POINT (2163741.043 11091938.871) |
| 211548 | 70.266777 | 21.816807 | asylchaos | POINT (2428635.902 11156105.608) |
| 211549 | 70.326849 | 30.646058 | herecomesthesun | POINT (3411503.516 11175940.376) |
| 211550 | 70.326849 | 30.646058 | migrants | POINT (3411503.516 11175940.376) |
| 211551 | 70.715060 | 23.454052 | asylbarn | POINT (2610893.181 11305540.881) |

211552 rows × 4 columns

### Note¶

The structure of the table **hashtag\_latlng** is a composite base based on the LBSN structure.

An SQL definition of **hashtag\_latlng** and all possible metrics and bases are provided here.

In [4]:

```
def create_grids():
    
    """
    Creating polygons based on the grid size
    """
    
    width = GRID_SIZE_METERS
    length = GRID_SIZE_METERS
    cols = list(range(int(np.floor(XMIN)), int(np.ceil(XMAX)), width))
    rows = list(range(int(np.floor(YMIN)), int(np.ceil(YMAX)), length))
    rows.reverse()

    polygons = []
    for x in cols:
         for y in rows:
                # combine to tuple: (x,y, poly)
                # and append to list
                polygons.append(
                    (x, y,
                     Polygon([
                         (x, y),
                         (x+width, y),
                         (x+width, y-length),
                         (x, y-length)])))
    grid = pd.DataFrame(polygons)
        # name columns
    col_labels=['xbin', 'ybin', 'bin_poly']
    grid.columns = col_labels
        # use x and y as index columns
    grid.set_index(['xbin', 'ybin'], inplace=True)
    grid = gp.GeoDataFrame(
            grid.drop(
                columns=["bin_poly"]),
                geometry=grid.bin_poly)
    grid.crs = CRS_PROJ
    return grid,cols,rows

grid,cols,rows = create_grids()
```

In [5]:

```
ybins = np.array(rows)
xbins = np.array(cols)

def get_best_bins(search_values_x, search_values_y,xbins, ybins): 
    """Will return best bin for a lat and lng input
    
    Note: prepare bins and values in correct matching projection
    
    """
    xbins_idx = np.digitize(search_values_x, xbins, right=False)
    ybins_idx = np.digitize(search_values_y, ybins, right=False)
    return (xbins[xbins_idx-1], ybins[ybins_idx-1])


xbins_match, ybins_match = get_best_bins(
    search_values_x=gdf.geometry.x.to_numpy(),
    search_values_y=gdf.geometry.y.to_numpy(),
    xbins=xbins, ybins=ybins)
```

In [6]:

```
"""
Assigning each hashtag with the results of bins. 
In this way we can use the bins as proxies for the polygons for faster calulations.
Additionally, the bin matches as well the index of the grid are being sorted to improve speed.

"""

df.loc[:, 'xbins_match'] = xbins_match
df.loc[:, 'ybins_match'] = ybins_match
df.drop(columns = ['longitude','latitude','geometry'],inplace =True)
df.set_index(['xbins_match', 'ybins_match'], inplace=True)
df.dropna(subset = 'hashtag', inplace =True)
grid.sort_index(inplace =True)
df.sort_index(inplace = True)
common_idx = grid.index.intersection(df.index) #instead of a spatial join, indexes are used to find which hashtag belongs to which grid
df
```

Out[6]:

|  |  | hashtag |
| --- | --- | --- |
| xbins\_match | ybins\_match |  |
| -1048668 | 3351138 | adolescenthealthemr |
|  | 3351138 | arabidol |
|  | 3351138 | atlasmountains |
|  | 3351138 | bfatun |
|  | 3351138 | can2017 |
| ... | ... | ... |
| 3851332 | 6851138 | immigrants |
|  | 6851138 | stpatricksday |
|  | 7251138 | refugeecrisis |
|  | 7251138 | leavingcert |
|  | 7251138 | worldrefugeeday |

211551 rows × 1 columns

In [23]:

```
#counting the occurence of each hashtag in preparation of typicality calculations
count = Counter()  
df['hashtag'] = df['hashtag'].str.lower()
df['hashtag'].str.split(',').apply(count.update)

#calculating frequency for total dataset     
n_t = count[HASHTAG]
N_t = sum(count.values())
F_t = n_t/N_t
```

In [8]:

```
def grid_typicality(new_test,idx):    
     
        #calculating frequency for each grid (sub-dataset) 
        counter = Counter()
        new_test.str.split(',').apply(counter.update)
        n_s = counter[HASHTAG]
        if (n_s == 0):
            typ.loc[idx,'typicality'] = -1.0
        else:    
            N_s = sum(counter.values())
            F_s = n_s/N_s
            typ.loc[idx,'typicality'] = (F_s - F_t)/F_t
```

In [24]:

```
typ = pd.DataFrame(index = common_idx, columns = ['typicality'], data = '') #dummy dataframe to hold the typicality values

for idx,midx in enumerate(common_idx): #looping through all the common indexes between the grids and dataframe
    grid_typicality(df.loc[midx,"hashtag"], common_idx[idx])
```

In [25]:

```
#creating a gdf of typicality per grid cell

geom = grid.loc[common_idx, "geometry"]
typ_gdf = gp.GeoDataFrame(data = typ['typicality'], geometry =geom, crs = CRS_PROJ)
typ_gdf
```

Out[25]:

|  |  | typicality | geometry |
| --- | --- | --- | --- |
| -1048668 | 3351138 | -1.0 | POLYGON ((-1048668.000 3351138.000, -948668.00... |
|  | 4451138 | -1.0 | POLYGON ((-1048668.000 4451138.000, -948668.00... |
|  | 4551138 | -1.0 | POLYGON ((-1048668.000 4551138.000, -948668.00... |
|  | 4651138 | -1.0 | POLYGON ((-1048668.000 4651138.000, -948668.00... |
|  | 4751138 | -1.0 | POLYGON ((-1048668.000 4751138.000, -948668.00... |
| ... | ... | ... | ... |
| 3751332 | 5651138 | -1.0 | POLYGON ((3751332.000 5651138.000, 3851332.000... |
| 3851332 | 4351138 | -1.0 | POLYGON ((3851332.000 4351138.000, 3951332.000... |
|  | 4751138 | -1.0 | POLYGON ((3851332.000 4751138.000, 3951332.000... |
|  | 6851138 | -1.0 | POLYGON ((3851332.000 6851138.000, 3951332.000... |
|  | 7251138 | -1.0 | POLYGON ((3851332.000 7251138.000, 3951332.000... |

1163 rows × 2 columns

In [26]:

```
classi = mc.UserDefined(typ_gdf['typicality'],[-1.0,-0.5,0.0,0.5, np.inf]) #user-defined classification scheme using map-classifier
mapping = dict([(i,s) for i,s in enumerate(classi.get_legend_classes())]) 

# creating the map
def replace_legend_items(legend, mapping): #function to change user-defined class intervals to values
    for txt in legend.texts:
        for k,v in mapping.items():
            if txt.get_text() == str(k):
                if str(v) == '[-1.00, -1.00]':
                    txt.set_text('-1.00')
                else:
                    txt.set_text(v[1:-1])

fig, ax =plt.subplots(1,1, figsize = (35,25))
 
typ_gdf.assign(cl = classi.yb).plot(column = 'cl',    
                                    cmap = CMAP,
                                    edgecolor ='gray',
                                    legend =True,
                                    categorical = True,
                                    alpha = 0.5,
                                    ax =ax)            
         

replace_legend_items(ax.get_legend(), mapping)
ctx.add_basemap(ax, crs=grid.crs.to_string(), source=ctx.providers.Stamen.TonerHybrid, alpha =0.6) #adding basemap for better context

plt.axis('off')

"""Uncomment the following line to save the plot"""

plt.savefig(f"{HASHTAG}.jpeg",pil_kwargs = {'quality' : 95, 'bbox_inches' : 'tight'})
```

### Working with HLL¶

As an example of preparation and analysis using HLL, these notebooks are available.

### Run this file locally¶

Follow the following instructions here to recreate the python environment. You can open this notebook once the environment is setup.
