# Supplementary material for: Analyzing the EU Migration Crisis as Reflected on Twitter
Source: KN J Cartogr Geogr Inf. 2022 Jul 9;72(3):213–28. doi: 10.1007/s42489-022-00114-6 (PMC9469823; doi:10.1007/s42489-022-00114-6)
Supplement: Supplementary file 10 — Supplementary file10 (HTML 820 KB) [file 42489_2022_114_MOESM10_ESM.html]

ESM\_2


# Analyzing the EU Migration Crisis as reflected on Twitter¶

### Jounral of Cartography and Geographic Information, Kartographische Nachrichten¶

Author Information

- **Sagnik Mukherjee** : sagnik.mukherjee1@tu-dresden.de ; https://orcid.org/0000-0001-8938-6154
- **Eva Hauthal** : eva.hauthal@tu-dresden.de ; https://orcid.org/0000-0001-8917-600X
- **Dirk Burghardt** : dirk.burghardt@tu-dresden.de ; https://orcid.org/0000-0003-2949-4887

Institute of Cartography, Technische Universität Dresden, 01069 Dresden, Germany

In [1]:

```
import warnings
warnings.filterwarnings('ignore')
from pathlib import Path
import pandas as pd
import geopandas as gp
from python_hll.hll import HLL
from python_hll.util import NumberUtil
from shapely.geometry import Point
import matplotlib.pyplot as plt
%matplotlib inline
import geoplot as gplt
import geoplot.crs as gcrs
import contextily as ctx


OUTPUT = Path.cwd().parents[0] / "Output"
df = pd.read_csv(Path.cwd().parents[0] /"CSV"/"HLL"/"ESM_3.csv",usecols = [0,1,2,3,4,5])
eu = gp.read_file(Path.cwd().parents[0]/ "Europe_coastline_shapefile" / "ESM_4.shp",crs = 'epsg:4326')
df['post_hll'] = df['post_hll'].apply(lambda x: x[2:])
df['date_hll'] = df['date_hll'].apply(lambda x: x[2:])
df['user_hll'] = df['user_hll'].apply(lambda x: x[2:])
df
```

Out[1]:

|  | user\_hll | post\_hll | date\_hll | latitude | longitude | hashtag |
| --- | --- | --- | --- | --- | --- | --- |
| 0 | 128b7fb4d8fa14cea9445b4a3eaa08e4f50475 | 128b7f81d7005ab2a56ed582a2d6fd191e353634077393... | 128b7fcb2e51821af86f9a019032cc0076d0dc39c8c9d3... | 28.017169 | 1.664273 | afrique |
| 1 | 128b7f9f066fa6767e1b74 | 128b7f1d27a3bfaaf36ec3 | 128b7fe6aa4fc35137c16a | 28.017169 | 1.664273 | alger |
| 2 | 128b7fff8583ff68e4d52c | 128b7fa09e9b1ed7ebcf78aad378f76965dcdc | 128b7f9f2a0a9344c7fd84 | 28.017169 | 1.664273 | algeria |
| 3 | 128b7fff8583ff68e4d52c | 128b7f3c1b3840c027340e | 128b7f56d8c1622eb9ba87 | 28.017169 | 1.664273 | algerian |
| 4 | 128b7fff8583ff68e4d52c | 128b7fa09e9b1ed7ebcf78aad378f76965dcdcdb15916a... | 128b7f9f2a0a9344c7fd84 | 28.017169 | 1.664273 | algerie |
| ... | ... | ... | ... | ... | ... | ... |
| 211547 | 128b7f118884db03390fed | 128b7f03feddaf4ede1ac3 | 128b7f4b1278ad70c8792b | 70.071230 | 19.437217 | refugeeswelcome |
| 211548 | 128b7fc0511d735a360ec4 | 128b7fc4a6567543b7ba85 | 128b7f4c59adaf6af1bdbc | 70.266777 | 21.816807 | asylchaos |
| 211549 | 128b7f3f848baabe36a655 | 128b7f697b9c7e91df9fde | 128b7f2cbcef3f1beacdc2 | 70.326849 | 30.646058 | herecomesthesun |
| 211550 | 128b7f3f848baabe36a655 | 128b7f697b9c7e91df9fde | 128b7f2cbcef3f1beacdc2 | 70.326849 | 30.646058 | migrants |
| 211551 | 128b7ff9d85f7adf62ac10 | 128b7f8ed8192757b191d0 | 128b7f91ada0f456cbfd1b | 70.715060 | 23.454052 | asylbarn |

211552 rows × 6 columns

In [3]:

```
df[df['hashtag']=='refugees']
```

Out[3]:

|  | user\_hll | post\_hll | date\_hll | latitude | longitude | hashtag |
| --- | --- | --- | --- | --- | --- | --- |
| 128 | 128b7fcc811bc230a04eeee1f87190d84dc218ff8583ff... | 128b7fb0a4c7eeb9d9ba77ddcee864ad217fd41734e241... | 128b7fb29f9436a045aa67ebedae6a9d0d2a3c1401a278... | 28.017169 | 1.664273 | refugees |
| 226 | 128b7fa13199b89bfe255b0dd3078f4376fa6d0f2ad833... | 128b7f9dc5232dbd5704aa598ceef859815cf976c21088... | 128b7fd8931f1b59033aa9517c399d91075130561286ee... | 28.326263 | -9.082743 | refugees |
| 239 | 128b7f267ba927e4f08874 | 128b7f5b9c9bb7032cd110 | 128b7f76c661ca9302e1e3 | 33.133300 | 11.216700 | refugees |
| 431 | 128b7f91a9899ebaf1281aa0fc48951db7b776a26f5fe2... | 128b7f838d45894abb2b87934cb916616adf95a2b00127... | 128b7f8b3764c365d5dcfd96a6dd6b81e47ebd9c9c77f4... | 33.894928 | 9.556354 | refugees |
| 533 | 128b7f7065dff9a30a2be2 | 128b7fe52341cdde638234 | 128b7fc05199fe6a5143dd | 35.141912 | 24.596244 | refugees |
| ... | ... | ... | ... | ... | ... | ... |
| 211437 | 128b7ffed999f137e50b86 | 128b7f75b58ceed3657d8f | 128b7fd55f943605c603a9 | 66.439735 | 14.360560 | refugees |
| 211455 | 128b7f14c7d8d7d03822da | 128b7f4eb61f291c5b3bbf | 128b7f75aacf112475ce34 | 66.669948 | 26.031108 | refugees |
| 211477 | 128b7f8a4a22a9d31ced0f | 128b7f30a4334fdc15f25c | 128b7f207430901888a654 | 67.295171 | 14.440035 | refugees |
| 211484 | 128b7fa1ba2f8c24ce23c9 | 128b7fd261434fd4cc0bee | 128b7f03733f0613767051 | 67.516345 | 13.694598 | refugees |
| 211528 | 128b7f04cf15fbb0f3e792 | 128b7f8f94ddf02c5794b1 | 128b7f92528cf62d2ced51 | 69.504585 | 29.727145 | refugees |

2943 rows × 6 columns

In [2]:

```
#hll functions

def hll_from_byte(hll_set):
    """Return HLL set from binary representation"""
     
    return HLL.from_bytes(
        NumberUtil.from_hex(
            hll_set, 0, len(hll_set)))


def union_hll(hll, hll2):
    """Union of two HLL sets. The first HLL set will be modified in-place."""
    hll.union(hll2)
    
def union_all_hll(hll_series,cardinality = True):
    """HLL Union and (optional) cardinality estimation from series of hll sets

        Args:
        hll_series: Indexed series (bins) of hll sets. 
        cardinality: If True, returns cardinality (counts). Otherwise,
            the unioned hll set will be returned.
    """
    
    hll_set = None
    for hll_set_str in hll_series.values.tolist():
        if hll_set is None:
            # set first hll set
            hll_set = hll_from_byte(hll_set_str)
            continue
        hll_set2 = hll_from_byte(hll_set_str)
        union_hll(hll_set, hll_set2)
    return hll_set.cardinality()
```

In [3]:

```
#union,intersection plot functions

def extract_tags(htag,keep_column):
    
    column_list = ['post_hll','user_hll','date_hll']
    column_list.remove(f'{keep_column}')
    mask =f"hashtag == '{htag}'"
    df_htag = df.query(mask)
    df_htag.drop(columns = column_list,inplace =True)
    return df_htag

def three_df(tag_a,tag_b,tag_c,column):
    
    df_a = extract_tags(f'{tag_a}',f'{column}')
    df_b = extract_tags(f'{tag_b}',f'{column}')
    df_c = extract_tags(f'{tag_c}',f'{column}')     
    calculate_post_total(df_a,df_b,df_c)
    set_operations(df_a,df_b,df_c)

def calculate_post_total(df_a,df_b,df_c):
    
    dfs = {
           f"{TAG_A}" : df_a,
           f"{TAG_B}": df_b,
           f"{TAG_C}" :df_c
           }

    ptotal = {}
    for hashtag, dfs in dfs.items():
        # drop bins with no values
        cardinality_total = union_all_hll(
            dfs[f"{COLUMN}"].dropna())
        ptotal[hashtag] = cardinality_total
        print(
            f"{ptotal[hashtag]} distinct posts "
            f"used {hashtag.upper()}")   
    d_common(df_a,df_b,df_c,ptotal) 
    
   
    
def d_common(df_a,df_b,df_c,ptotal):
    
    union_a_b = pd.concat([df_a, df_b])
    union_a_c = pd.concat([df_a, df_c])
    union_b_c = pd.concat([df_c, df_b])


    dfs = {
        f"{TAG_A}_{TAG_C}": union_a_c,
        f"{TAG_A}_{TAG_B}": union_a_b,
        f"{TAG_B}_{TAG_C}": union_b_c
    }
    distinct_common = {}
    for hashtag_set, dfs in dfs.items():
        cardinality = union_all_hll(
            dfs[f"{COLUMN}"].dropna())
        distinct_common[hashtag_set] = cardinality
        print(
            f"{distinct_common[hashtag_set]} distinct total posts "
            f"which had either {hashtag_set.split('_')[0]} "
            f"or {hashtag_set.split('_')[1]} (union)")
        
    distinct_intersection = {}
    for a, b in [(f"{TAG_A}", f"{TAG_B}"), (f"{TAG_A}", f"{TAG_C}"), (f"{TAG_B}", f"{TAG_C}")]:
        a_total = ptotal[a]
        b_total = ptotal[b]
        common_tags = f'{a}_{b}'
        intersection_count = a_total + b_total - distinct_common[common_tags]
        distinct_intersection[common_tags] = intersection_count
        print(
            f"{distinct_intersection[common_tags]} distinct posts "
            f"with hashtags with {a} and {b} (intersection)")
     
    union_a_b_c = pd.concat([df_a, df_b, df_c])
    cardinality = union_all_hll(
        union_a_b_c[f"{COLUMN}"].dropna())
    union_count_all = cardinality    
    intersection_count_all = union_count_all - \
    ptotal[TAG_A] - \
    ptotal[TAG_B] - \
    ptotal[TAG_C] + \
    distinct_intersection[f'{TAG_A}_{TAG_B}'] + \
    distinct_intersection[f'{TAG_A}_{TAG_C}'] + \
    distinct_intersection[f'{TAG_B}_{TAG_C}']
    
    print(f'Union Count : {union_count_all}',
          f'Intersection Count : {intersection_count_all}')
    
def make_tuple(lat,lon):
    return (lat,lon)    

def make_lists(dfx):
    return dfx.apply(lambda x: make_tuple(x.latitude,x.longitude),axis=1).tolist()

def set_operations(df_a,df_b,df_c):
    set_a = set(make_lists(df_a))
    set_b = set(make_lists(df_b))
    set_c = set(make_lists(df_c))
    
    union_final = set_a.union(set_b,set_c)
    
    intersect_foo = set(set_a).intersection(set_b)
    intersect_final = set(set_c).intersection(intersect_foo)
    
    makeGeoSeries(union_final,intersect_final)

def makeGeoSeries(union, intersect):
    
    union_foo = [Point(coord[1], coord[0]) for coord in union]
    union_points = gp.GeoSeries(union_foo)
    union_points.set_crs(epsg =3857,inplace =True)
    
    intersect_foo = [Point(coord[1], coord[0]) for coord in intersect]
    intersect_points = gp.GeoSeries(intersect_foo)
    intersect_points.set_crs(epsg =3857,inplace =True)
    
    plot(union_points,intersect_points)
    
def plot(u_points,i_points):
    
    union = u_points
    intersection = i_points
    
    fig,axes = plt.subplots(ncols =2,
                          nrows =1,
                          figsize=(20,10),
                          subplot_kw={'projection': gcrs.WebMercator()}
                           )
    gplt.webmap(eu, projection=gcrs.WebMercator(),
                provider = ctx.providers.Stamen.TonerLite,
                ax =axes[0]
                     )
    gplt.kdeplot(union, ax=axes[0],n_levels=15, cmap='turbo')
    axes[0].set_title("KDE of union function points",fontsize =10)
    
                             
    gplt.webmap(eu, projection=gcrs.WebMercator(),
                provider = ctx.providers.Stamen.TonerLite,
                ax =axes[1]
                     )
    gplt.kdeplot(intersection, ax=axes[1],n_levels=15, cmap='turbo')
    axes[1].set_title("KDE of intersection function points",fontsize =10)
    plt.savefig(OUTPUT / 'USERHLL.jpeg',quality =95,bbox_inches = 'tight')
```

In [4]:

```
%%time

TAG_A ='refugees'
TAG_B = 'refugeeswelcome'
TAG_C = 'migrants'
COLUMN = "user_hll"

three_df(TAG_A,TAG_B,TAG_C,COLUMN)
```

```
6784 distinct posts used REFUGEES
5743 distinct posts used REFUGEESWELCOME
3907 distinct posts used MIGRANTS
9627 distinct total posts which had either refugees or migrants (union)
11151 distinct total posts which had either refugees or refugeeswelcome (union)
9130 distinct total posts which had either refugeeswelcome or migrants (union)
1376 distinct posts with hashtags with refugees and refugeeswelcome (intersection)
1064 distinct posts with hashtags with refugees and migrants (intersection)
520 distinct posts with hashtags with refugeeswelcome and migrants (intersection)
Union Count : 13666 Intersection Count : 192
CPU times: user 54.2 s, sys: 231 ms, total: 54.4 s
Wall time: 1min
```

For further Information, please check the following links:

HLL Operations with YFCC 100m dataset : https://ad.vgiscience.org/yfcc\_gridagg/01\_preparations.html

HLL Operations with Instagram Sunrise-Sunset dataset : https://wwwpub.zih.tu-dresden.de/~s7398234/vis/instagram/Instagram\_sunrise\_sample.html

General Information on LBSN: https://lbsn.vgiscience.org/
